# *Epichloë* Fungal Endophytes Influence Seed-Associated Bacterial Communities

**DOI:** 10.3389/fmicb.2021.795354

**Published:** 2022-01-04

**Authors:** Daniel A. Bastías, Ludmila Bubica Bustos, Ruy Jáuregui, Andrea Barrera, Ian S. Acuña-Rodríguez, Marco A. Molina-Montenegro, Pedro E. Gundel

**Affiliations:** ^1^AgResearch Limited, Grasslands Research Centre, Palmerston North, New Zealand; ^2^IFEVA, CONICET, Facultad de Agronomía, Universidad de Buenos Aires, Buenos Aires, Argentina; ^3^Laboratorio de Ecología Integrativa, Instituto de Ciencias Biológicas, Universidad de Talca, Talca, Chile; ^4^Centro de Estudios Avanzados en Zonas Áridas (CEAZA), Universidad Católica del Norte, Coquimbo, Chile; ^5^Centro de Investigación de Estudios Avanzados del Maule, Universidad Católica del Maule, Talca, Chile

**Keywords:** seed microbiota, plant-associated bacterial communities, *Epichloë* endophytes, plant-microbe interactions, herbivory

## Abstract

Seeds commonly harbour diverse bacterial communities that can enhance the fitness of future plants. The bacterial microbiota associated with mother plant’s foliar tissues is one of the main sources of bacteria for seeds. Therefore, any ecological factor influencing the mother plant’s microbiota may also affect the diversity of the seed’s bacterial community. Grasses form associations with beneficial vertically transmitted fungal endophytes of genus *Epichloë*. The interaction of plants with *Epichloë* endophytes and insect herbivores can influence the plant foliar microbiota. However, it is unknown whether these interactions (alone or in concert) can affect the assembly of bacterial communities in the produced seed. We subjected *Lolium multiflorum* plants with and without its common endophyte *Epichloë occultans* (E+, E-, respectively) to an herbivory treatment with *Rhopalosiphum padi* aphids and assessed the diversity and composition of the bacterial communities in the produced seed. The presence of *Epichloë* endophytes influenced the seed bacterial microbiota by increasing the diversity and affecting the composition of the communities. The relative abundances of the bacterial taxa were more similarly distributed in communities associated with E+ than E- seeds with the latter being dominated by just a few bacterial groups. Contrary to our expectations, seed bacterial communities were not affected by the aphid herbivory experienced by mother plants. We speculate that the enhanced seed/seedling performance documented for *Epichloë*-host associations may be explained, at least in part, by the *Epichloë*-mediated increment in the seed-bacterial diversity, and that this phenomenon may be applicable to other plant-endophyte associations.

## Introduction

Plant seeds commonly harbour a diverse community of microbes including bacteria and fungi (Nelson, 2018). The seed-associated microbes are generally critical for plant fitness since they can confer protection against herbivores and pathogens, increase germination, enhance seedlings growth, and increase plant tolerance to stresses (Truyens et al., 2015; Bastias et al., 2017; Rochefort et al., 2021). Members of the seed microbiota can be acquired from different sources namely certain mother plant tissues, plant reproductive structures and the environment (Shade et al., 2017; Fort et al., 2021). Those that are inherited from mother plants are termed as ‘vertically transmitted microbes’, whereas those recruited from the external environment are termed as ‘horizontally transmitted microbes’ (Shade et al., 2017). Since the mother plant’s microbiota is one of the main sources of microbes for seeds, any ecological factor influencing this source could eventually affect the diversity and composition of the seed’s microbial community (Chesneau et al., 2020; Fort et al., 2021). In fact, it is well documented that plant leaf microbial communities can be influenced by the interaction of plants with beneficial persistent microorganisms and herbivores (Nissinen et al., 2019; Seabloom et al., 2019; Humphrey and Whiteman, 2020). However, it is not fully understood whether the effects of these ecological factors on the mother plant microbiota can persist and affect the assemblies of microbes associated with the produced seeds (Wei and Jousset, 2017). A remarkable symbiotic system to evaluate the effects of ecological interactions experienced by mother plants on seed-associated microbial communities is the association between cool-season grasses and asexual fungal endophytes of the genus *Epichloë*. These endophytes are persistent symbionts of grasses (they grow in foliar host tissues during the entire plant life) and are vertically transmitted through the seeds (Schardl et al., 2004). Because *Epichloë* endophytes confer antiherbivore protection and can modulate the leaf microbiota of mother plants (e.g., Bastias et al., 2017; Nissinen et al., 2019), we hypothesise that these symbionts may also influence the diversity and composition of the seed microbiota.

The potential effects of *Epichloë* endophytes on the diversity and composition of seed microbial communities have been barely explored (e.g., Tannenbaum et al., 2021). *Epichloë* may affect the seed microbiota though its effects on the microbial assemblies of mother plants. For example, the presence of *Epichloë coenophiala* endophytes modified the leaf-associated fungal communities in *Festuca arundinacea* plants (Syn. *Schedonorus phoenix*; Nissinen et al., 2019). In addition, *Epichloë festucae* var. *lolii* endophytes re-organised the bacterial communities associated with seedlings of *Lolium perenne* (Tannenbaum et al., 2020). *Epichloë* symbionts may also affect the seed microbiota *via* the transport of certain endophytic microbes into the developing seed. At the host reproductive stage, *Epichloë* extend hyphae into fertilised ovaries and later, into embryos and other seed structures as part of the vertical transmission process (Gundel et al., 2011b; Liu et al., 2017). Therefore, microbes located on fungal hyphae of *Epichloë* spp. could eventually be transmitted to seeds. In fact, bacteria inhabiting the external surface of *Epichloë* mycelia have been recently isolated (Bastías et al., 2020). The potential regulation of the seed microbiota by *Epichloë* endophytes could also arise due to *Epichloë*-mediated changes on seed chemical composition. *Epichloë* endophytes can modify the concentrations of sugars (i.e., mannitol, ribitol and thehalose) and antioxidants (i.e., tocochromanols and glutathione) in seeds (Gundel et al., 2018; Zhang et al., 2019; Hettiarachchige et al., 2021). In addition, the accumulation of *Epichloë*-derived alkaloids has been shown to modulate bacterial communities associated with host plant leaves (Roberts and Lindow, 2014) and as such may also influence the seed microbiota (Hewitt et al., 2020). *Epichloë* could also modify the seed microbial composition *via* competition for plant resources. The successful competition for plant resources by *Epichloë occultans* endophytes seemed to exclude the pathogen *Claviceps purpurea* from *Lolium multiflorum* plant flowers (Pérez et al., 2013).

The interaction of mother plants with herbivores can also affect their foliar-associated microbial communities and, eventually, the microbiota of their produced seed. For example, the bacterial diversity in the leaves of *Cardamine cordifolia* plants was modified by the attack of the foliar chewing insect *Scaptomyza nigrita* (Humphrey and Whiteman, 2020). The herbivore-mediated changes in leaf plant microbiota could be explained by the activation of plants defences (acting negatively on microbes), the release of plant nutrients from damaged tissues, and the inoculation of microbes from the herbivore into plant tissues (e.g., microbes inhabiting its oral secretions; Smets and Koskella, 2020). In the case of the interaction between *C. cordifolia* plants and *S. nigrita* insects, the plant activation of defences responses due to herbivore attack (i.e., activation of jasmonic acid-dependent responses), explained the changes in bacterial diversity (Humphrey and Whiteman, 2020). Interestingly, since *Epichloë* spp. can reduce herbivore pressure on plants, due to the production of anti-herbivore alkaloids (Bastias et al., 2017), the effects of herbivores on the mother plant’s microbiota (and eventually on their produced seeds) might be reduced in *Epichloë* infected associations. In regard to plant defences, it is important to underscore that *Epichloë* can also modulate host plant immune responses (i.e., salicylic acid and jasmonic acid signalling pathways; Bastias et al., 2017). This endophyte-modulation of host plant immunity could affect directly the mother plant’s microbiota *via* excluding microbial groups (Shi et al., 2020; Kou et al., 2021), and/or indirectly though changing the levels of plant resistance to herbivores (e.g., Bastías et al., 2018).

Here, we studied the effects of the interaction between *Epichloë* endophytes and insect herbivory experienced by mother plants on the diversity and composition of seed-associated bacterial communities. We subjected mother plants of *L. multiflorum* (common name: Italian ryegrass) with and without its common endophyte *E. occultans* to a challenge with *Rhopalosiphum padi* aphids to further characterise the bacterial communities associated with seed produced by these plants. This aphid species commonly interacts with the grass *L. multiflorum* in natural grasslands and cultivated pastures (Omacini et al., 2001; Weibull, 1993). *Epichloë occultans* is a vertically transmitted fungus that produces loline alkaloids, compounds with known bioactivity against *R. padi* aphids (Bastías et al., 2017). Due to the documented effects of *Epichloë* endophytes on the mother plant-associated microbiota and seed biochemical composition, we hypothesise that seed-associated bacterial microbiota will be influenced by the *Epichloë* presence. We also hypothesise that assemblies of seed-associated bacterial communities will vary due to the herbivory history of mother plants. However, since *Epichloë* endophytes also modulate plant-herbivore interactions, the potential effect of the herbivory experienced by mother plants on seed-associated bacterial communities may be attenuated.

## Materials and Methods

### Biological Material

We used the symbiotic interaction between *L. multiflorum* and the fungal endophyte *E. occultans* as our study system. *Lolium multiflorum* is an annual grass native to Mediterranean region (currently worldwide distributed in ecosystems with temperate humid climates), and naturally associated with the fungal endophyte *E. occultans* (Moon et al., 2000). The vertical transmission efficiency of this endophyte species is usually high (close to 100%; Gundel et al., 2011a).

In our group, we maintain two plant biotypes for experimentation, endophyte-symbiotic (E+) and endophyte-free (E-) plants. Endophyte free plants were initially obtained by removing the endophyte from naturally E+ plants with a systemic fungicide (Gundel et al., 2012). Since then, we have been cultivating every year E+ and E- plants as to produce fresh seeds for experiments. We grow and maintain 25 E+ and 25 E- plants during the whole growing cycle to produce seeds. The plants are randomly sowed on a square plot (distance between plants: 0.50 m). The plot is maintained clean from weeds, and plants are watered on demand. Biotypes are maintained as true to type since *E. occultans* is only vertically transmitted through the seed (this endophyte species cannot be horizontally transmitted between plant biotypes; Gundel et al., 2011b). Furthermore, *L. multiflorum* is a self-incompatible and wind-pollinated species, thus the exchange of pollen between plants at flowering avoids the genetic differentiation between the E+ and E- biotypes (Gundel et al., 2012). Ripened seeds from each individual plant are harvested every year and the endophytic status assessed (using the ‘seed squash’ technique) prior to generate E+ and E- pools of seed for experimentation (Card et al., 2011).

We used the generalist phytophagous herbivore aphid *R. padi* L. (Hemiptera; Aphididae) for experimentation. This aphid species is common pest in cereals and grasses (Dixon, 1971; van Emden and Harrington, 2017). *Rhopalosiphum padi* aphids were collected from volunteer plants, transferred to Petri plates containing leaves of *Avena sativa* L., and maintained for 5 days to eliminate parasitised insects. Apterous adults free of parasites were placed on young *A. sativa* plants to initiate the insect population for experimentation. Plants were periodically watered and constantly replaced by healthy ones. The aphid populations were grown under optimal controlled conditions [temperature: 23°C (±1), photoperiod: L16-D8 h, radiation: 150 μmol m^−2^ s^−1^].

### Experimental Design

We sowed six E+ and six E- plants. Each *L. multiflorum* plant was individually grown in 3 L pots filled with a commercial potting mix substrate. The experiment was carried out outdoors (at spring season), placing plants on 1-m-high benches, and maintaining 0.2 m of distance between pots. One month after sowing, three plants of each symbiotic biotype were randomly assigned to any of the following treatments: herbivore-free (H-) or herbivory-challenged (H+) with *R. padi* aphids. Plants were at tillering stage with 6–10 tillers each. In the herbivory treatment, 10 apterous adult aphids were placed on each plant, and resulting populations were allowed to grow for 21 days. Each plant was individually enclosed with a tubular plastic net (0.05 mm mesh) to maintain the aphids under confinement, and to avoid the entrance of any external insect. At day 21, aphids and tubular nets were removed from the experimental plants, and these plants continued growing in the same conditions (on benches, outdoors) until the end of the growing cycle (they were periodically inspected to avoid water deficit and pest attacks). At flowering stage, plants were allowed to exchange pollen freely (with experimental and surrounding plants). At the end of the growing cycle, plants were characterised measuring dry weight (DW) shoot biomass, seed production, and the *Epichloë* presence within the produced seed. Shoot plant biomass was determined after drying the foliar tissues in an oven (60–70°C) for 48 h. Mature seeds were harvested from each mother plant and placed in paper bags. All the seed bags (*n* = 12) were stored at 4°C in plastic boxes containing silica gels until DNA extraction (see below). Endophyte presence was determined by inspecting 10 seeds per mother plant using the ‘seed squash’ technique (Card et al., 2011).

### Characterisation of Seed-Associated Bacterial Communities

#### DNA Extraction, Library Preparation and Sequencing

Bacterial communities were characterised by sequencing the bacterial 16S ribosomal RNA (rRNA) gene though Illumina MiSeq sequencer (paired end, 2x300bp; Illumina, United States). For the DNA extraction, 25 seeds were randomly selected from each seed lot. Seeds were surface sterilised with 70% ethanol solution (Ma et al., 2015), and homogenised with sterile ceramic beads using a TissueLyser II (Qiagen, United States). DNA was extracted on 50 mg of homogenised seed material using the GenElute Plant Genomic DNA Miniprep kit (Sigma-Aldrich, United States) following manufacturer’s instructions. Samples were processed under laminar flow chambers to avoid environmental contamination. DNA integrity was assessed by electrophoresis on 1% agarose gels while DNA purity and concentration were checked with a Nanodrop spectrophotometer (Nanodrop Technologies, United States). Libraries for sequencing were prepared by Macrogen Inc. (Seoul, South Korea) using universal primers targeting the V3/V4 region within the 16S rRNA gene sequence (primers 341F: CCTACGGGNGGCWGCAG; 805R: GACTACHVGGGTATCTAATCC). These primers have been frequently used in bacterial metabarcoding studies (Herlemann et al., 2011).

#### Processing of Sequencing Data

The sequencing instrument produced ~1.22 million read pairs with an average of 102 K read pairs per sample. The amplicon reads were processed using a modified pipeline from Camarinha-Silva et al. (2012). The sequence reads were paired using the program FLASH2 (Magoč and Salzberg, 2011), and the paired reads were quality trimmed using Trimmomatic (Bolger et al., 2014). The trimmed reads were reformatted into FASTA, and read headers were modified to include sample names. All reads were compiled in a single file. The Mothur program suit was used to remove reads with homopolymers longer than 10 nucleotides, and to collapse the reads into unique representatives (Schloss et al., 2009). The Swarm program was used to cluster the collapsed reads (Mahé et al., 2014). The clustered reads were filtered based on their abundances, maintaining the reads that were (a) present in one sample with a relative abundance >0.1%, (b) present in >2% of the samples with a relative abundance >0.01% or (c) present in 5% of the samples at any abundance level. The selected reads were annotated using the QIIME program (version 1.9.1) with the Silva database (version 138; Caporaso et al., 2010; Quast et al., 2013). A Ruby program that implements the above-described abundance filter is available in the AgResearch Gitea website: https://gitea.agresearch.co.nz/JAUREGUIR/Microbiomics. The taxonomic affiliation of each operational taxonomic units (OTU) was verified using the Ribosomal Database Project (RDP) classifier and the 16S rRNA training set 18 as reference database (Wang et al., 2007). OTUs from chloroplast or mitochondrial plant sequences were removed from the database prior to analyses.

The final table included 99 bacterial OTUs representing 282,784 high-quality sequence reads (see Supplementary Table S1). All rarefaction curves associated with seed samples reached asymptotes (number of OTUs vs. number of sequencing reads) (Supplementary Figure S1), indicating that the sequencing depth described most of the bacterial assemblages (Wooley et al., 2010).

A phylogenetic tree was constructed for dominant seed-associated bacterial taxonomic groups using MEGA X software (Kumar et al., 2018). The sequence alignments were performed with MAFFT in Geneious software using default parameters (Kearse et al., 2012). Phylogenetic reconstruction was carried out using the Unweighted Pair Group Method with Arithmetic mean (UPGMA) algorithm with a bootstrap test (9,999 replicates) and default parameters (Hall, 2013).

### Statistical Analyses

The effect of the plant symbiosis status and the herbivory history on the response variables ‘plant shoot biomass’, ‘seed production’, ‘bacterial abundance (number of reads)’, ‘species richness (number of OTUs)’, ‘species evenness’ and ‘Shannon diversity’ of the OTUs were analysed using linear effect models with the *gls* function from the *nlme* R package (Pinheiro et al., 2009). We assumed independent, identically distributed normal random errors. The models included the plant’s symbiotic status (E+, E-) and herbivory history (H+, H-) as categorical factors. VarIdent variance structures were used on the interaction between symbiotic status and herbivory history to accommodate deviations in variance homogeneity in the bacterial abundance response variable. Assumptions of each analysis of variance were met before to perform the analyses.

The effect of the plant symbiosis status and the herbivory history on the composition of bacterial assemblages of plant seed were analysed using permutational multivariate analysis of variance (PERMANOVA; 9,999 permutations), based on Bray-Curtis distances, with the function *adonis* from the *vegan* R package (Anderson, 2001; Oksanen et al., 2020). PERMANOVA allows testing for differences between groups when multivariate responses are measured. Similarly, the model included the plant’s symbiotic status (E+, E-) and herbivory history (H+, H-) as categorical factors. To characterise the composition differences between bacterial assemblages associated with seed, non-metric multidimensional scaling (NMDS) based on Bray-Curtis dissimilarities were performed using the *vegan* R package (Anderson, 2001; Oksanen et al., 2020). A Shepard diagram correlating NMDS ordination distances and Bray-Curtis community dissimilarities was used to validate NMDS ordination analysis. A high correlation indicates that the calculated NMDS ordination is a good representation of community structures (Leeuw and Mair, 2015). All the presented values in the result section are means ± standard errors (S.E.M). All the analyses were performed on R software (version 3.1.1; R Core Team, 2014).

## Results

The plant shoot biomass was not affected by either the plant symbiosis status (*F*_(1,8)_ = 2.78, *p* = 0.134) or the aphid herbivory (F_(1,8)_ = 0.00, *p* = 0.978). E+ plants marginally produced more seed than E- plants (F_(1,8)_ = 4.96, *p* = 0.05). The aphid herbivory did not affect the plant seed production (F_(1,8)_ = 0.04, *p* = 0.835). All seed produced by E+ plants contained *Epichloë* endophytes, whereas seed from E- plants were free of these endophytes. The aphid herbivory treatment did not affect the *Epichloë* seed transmission (Table 1).

**Table 1:** Shoot biomass, seed production, seed number and endophyte seed transmission in *Lolium multiflorum* plants symbiotic (E+) and non-symbiotic (E-) with the fungal endophyte *Epichloë occultans*, that were challenged (H+) or not (H-) with *Rhopalosiphum padi* aphids.

Symbiosis/herbivory	Shoot plant biomass (g DW ∙ plant^−1^)	Seed production (g ∙ plant^−1^)	Seed number (# ∙ plant^−1^)	*Epichloë* seed transmission (%)
E-/H-	12.52 ± 0.66a	2.53 ± 0.36a	~1372.07	0
E+/H-	12.06 ± 0.91a	2.89 ± 0.33b	~1386.76	100
E-/H+	12.37 ± 1.58a	1.64 ± 0.25a	~890.09	0
E+/H+	13.04 ± 1.03a	3.48 ± 0.26b	~1668.26	100

Different letters in values indicate significant differences (*p* < 0.050). Endophyte transmission was determined by inspecting 10 seeds per mother plant. Values are means ± SEM (*n* = 3 plants).

**Figure 1:**
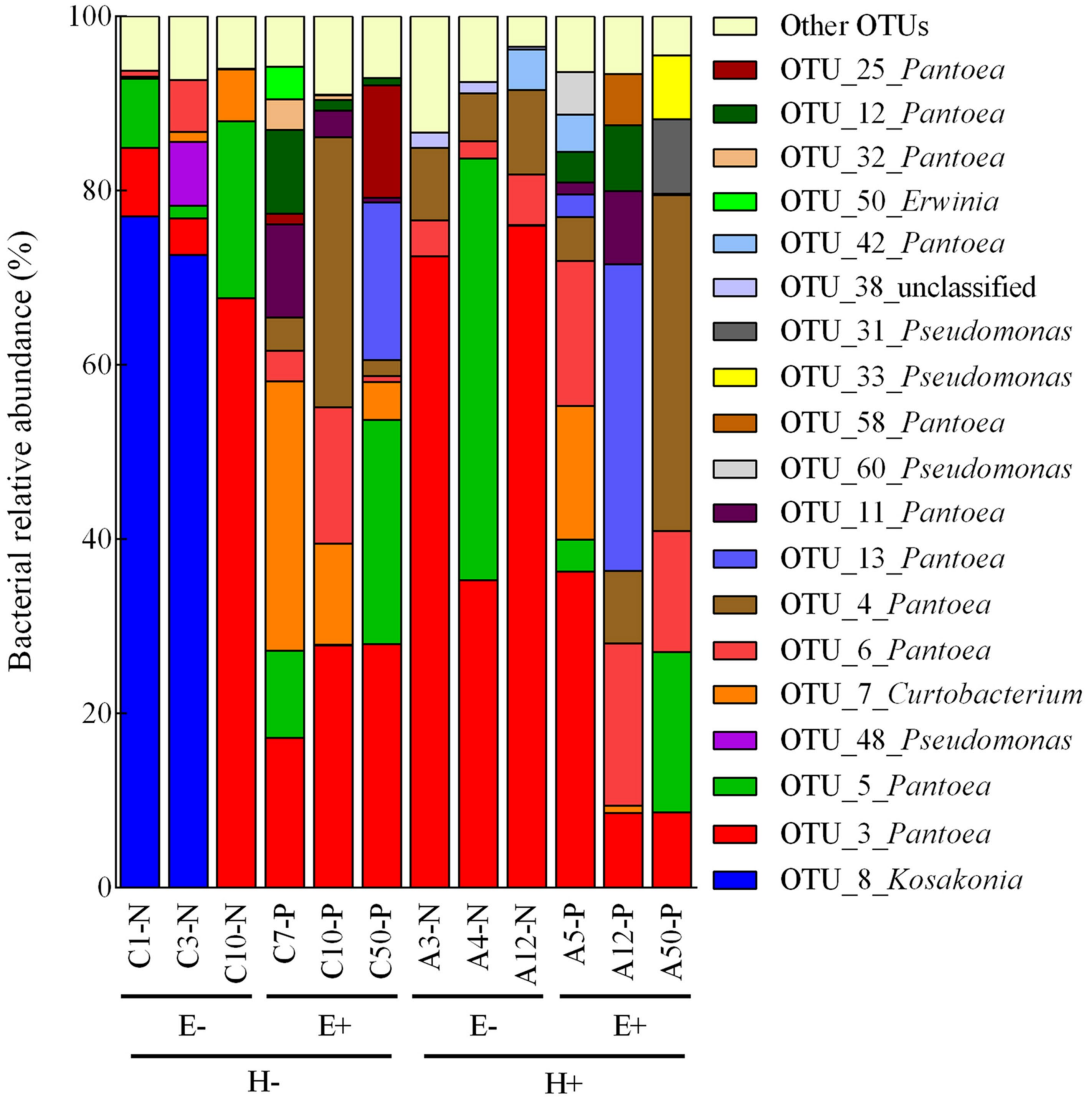
Relative abundance of dominant bacterial OTUs (operational taxonomic units) associated with individual seed samples of *Lolium multiflorum* plants symbiotic (E+) or not (E-) with the fungal endophyte *Epichloë occultans*, and challenged (H+) or not (H-) with the aphid herbivore *Rhopalosiphum padi*. Dominant bacterial OTUs were those with relative abundances above 1%, on average, in at least one group (i.e., E-/H-, E+/H-, E+/H- and E+/H+). Dominant bacterial OTUs represented 86-95% of the abundances of sequence reads associated with each seed sample. OTUs with relative abundances below 1% were compiled in the ‘other OTUs’ group.

We identified 99 bacterial OTUs in seed produced by *L. multiflorum* plants, with an average of 56.58 ± 2.11 OTUs per seed sample (i.e., from a mother plant). These bacterial OTUs were identified in phyla Proteobacteria (74% of OTUs), Actinobacteria (13% of OTUs), and Firmicutes (4% of OTUs; the remaining 9% of OTUs could not be classified at the phylum level; Supplementary Table S1). Considering the experimental conditions (i.e., endophyte presence within seed and plant herbivory history), bacterial assemblages were dominated by 6–13 OTUs (relative abundances > 1%; number of OTUs: E-/H- = 6, E-/H+ = 6, E+/H- = 11, E+/H+ = 13; Figure 1). Mostly these OTUs were identified in phylum Proteobacteria (i.e., *Pantoea*, *Pseudomonas*, *Erwinia* and *Kosakonia*) with only one belonging to Actinobacteria (i.e., *Curtobacterium*; Figure 1).

**Figure 2:**
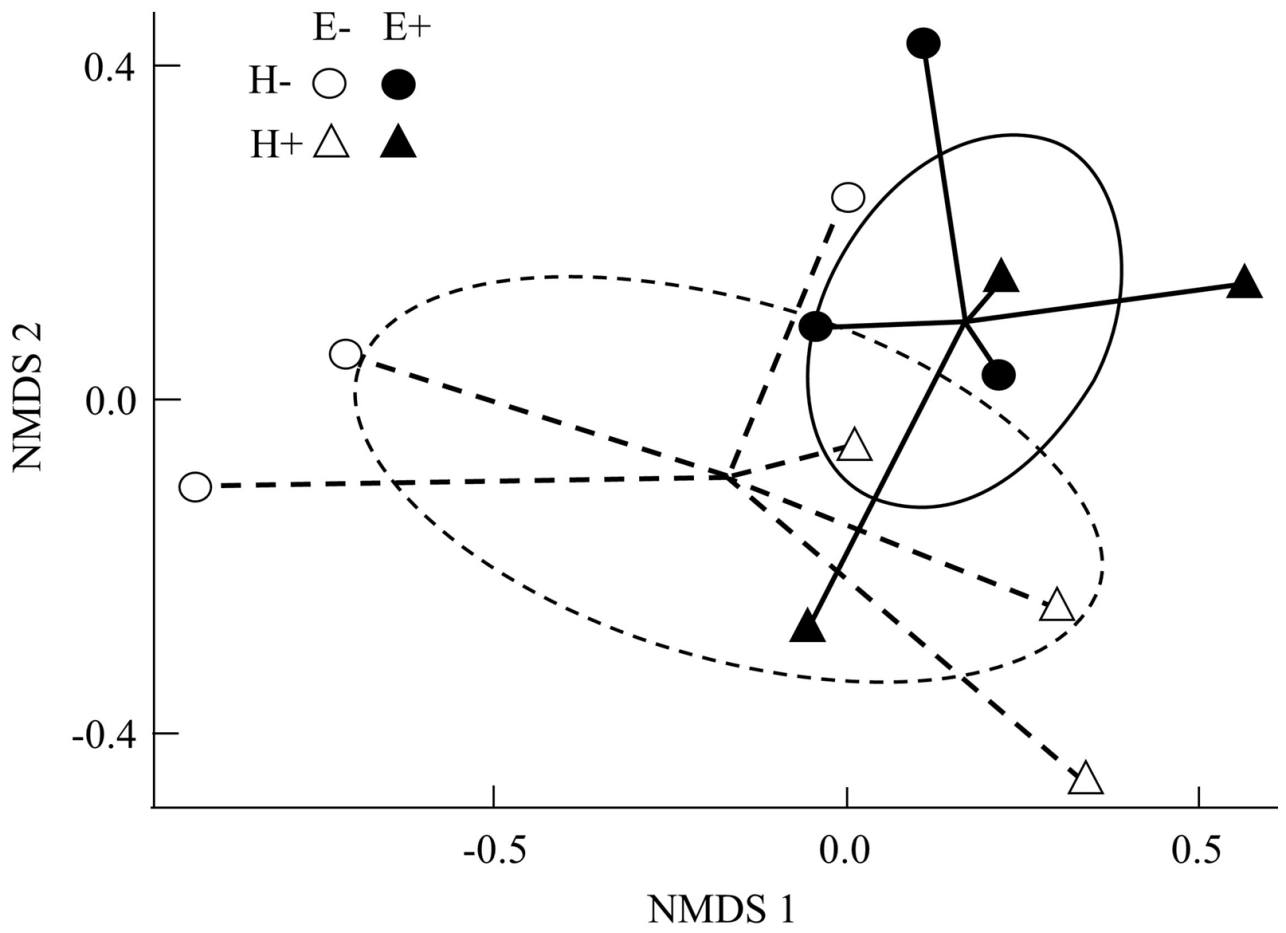
Non-metric multidimensional scaling ordinations (NMDS; stress = 0.09) for bacterial assemblages associated with seed of *Lolium multiflorum* plants symbiotic (E+, filled symbols) or not (E-, open symbols) with the fungal endophyte *Epichloë occultans*, and challenged (H+, triangles) or not (H-, circles) with the aphid herbivore *Rhopalosiphum padi*. Lines indicate compositional distances between centroids and E+ and E- seed-associated bacterial communities (continuous and discontinuous lines, respectively). Ellipses represent 95% confidence intervals around centroids and show clustering of bacterial compositions in seed based on the presence/absence of *Epichloë* endophytes (continuous and discontinuous lines, respectively). The Shepard diagram (Supplementary Figure S2) showed a good linear fit between ordination distances and Bray-Curtis dissimilarities (*n* = 3 plants).

**Figure 3:**
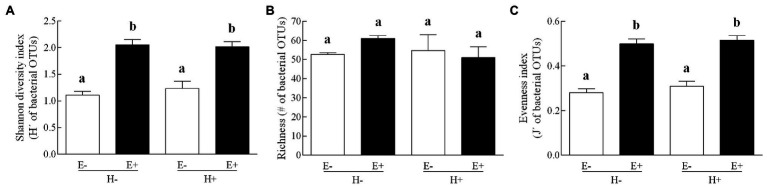
Bacterial diversity indexes (panel **A**: Shannon index; panel **B**: richness; panel **C**: evenness index) associated with seed of *Lolium multiflorum* plants symbiotic (E+, filled bars) or not (E-, open bars) with the fungal endophyte *Epichloë occultans*, and challenged (H+) or not (H-) with the aphid herbivore *Rhopalosiphum padi*. OTUs means operational taxonomic units. Different letters indicate significant differences at *p* < 0.050. Bars represent means values ± SEM (*n* = 3 plants).

**Figure 4:**
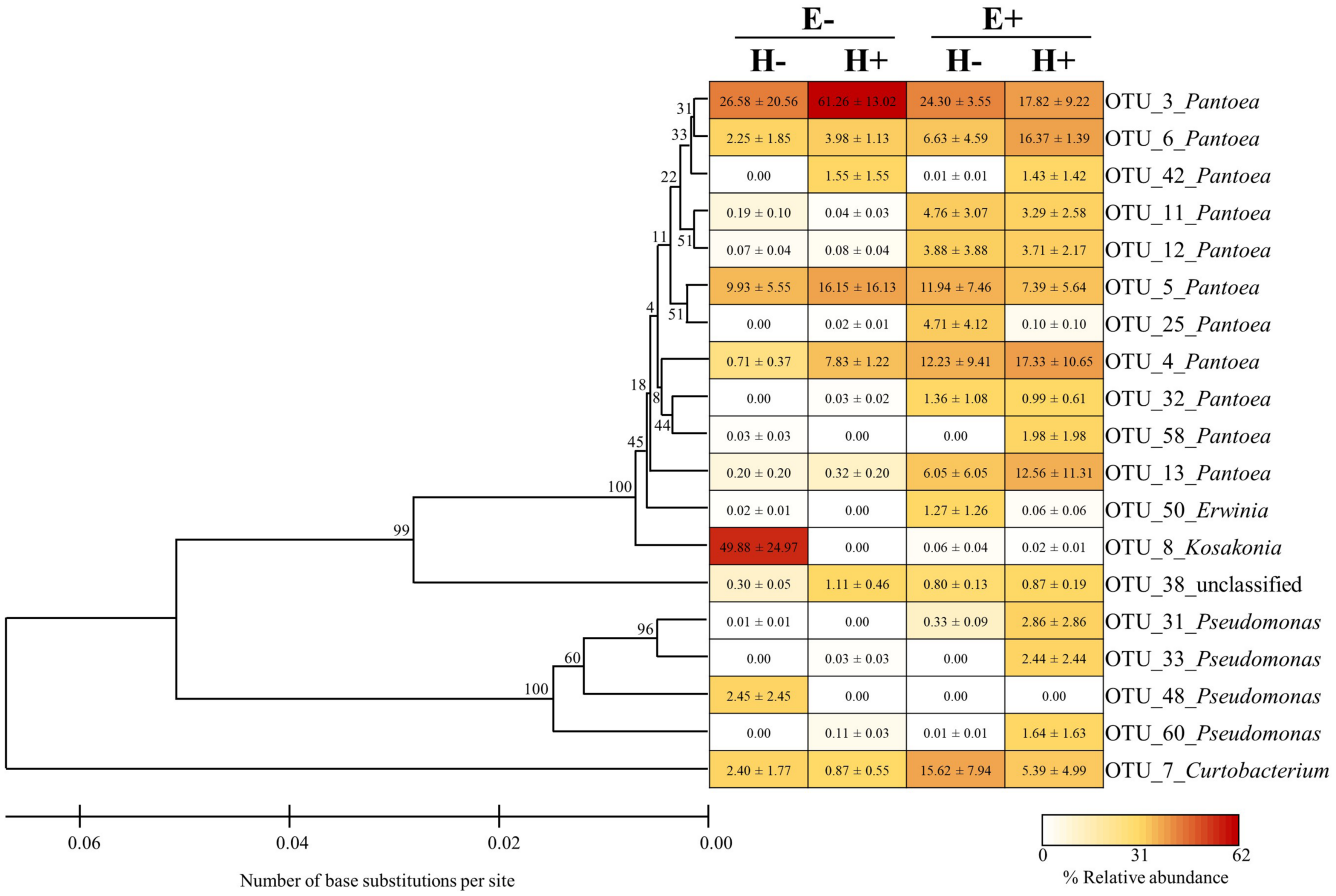
Phylogenetic tree and relative abundance of bacterial OTUs (operational taxonomic units) associated with seed of *Lolium multiflorum* plants symbiotic (E+) or not (E-) the fungal endophyte *Epichloë occultans*, and challenged (H+) or not (H-) with the aphid herbivore *Rhopalosiphum padi*. The tree was inferred using the Unweighted Pair Group Method with Arithmetic mean (UPGMA) algorithm with a bootstrap test (9,999 replicates). Bootstrap values for ingroup clades are shown next to branches. Evolutionary distances were calculated using the Maximum Composite Likelihood method. The heat map represents the relative abundances of dominant bacterial OTU associated with each seed group. Dominant bacterial OTUs (>1% relative abundance) represented 91-94% of the abundances of sequence reads associated with each seed group. Values in cells indicate means ± SEM (*n* = 3 plants).

The composition of the seed bacterial assemblages differed between endophyte-symbiotic and endophyte-free *L. multiflorum* plants, but it was independent on the plant herbivory history (Table 2). This was visualised with a NMDS ordination that showed that bacterial communities were mainly grouped by the symbiotic status of seeds rather than by the maternal herbivory history (Figure 2). The Shannon diversity index increased *ca*. 42% by the endophyte presence (E+ = 2.03 ± 0.06, E- = 1.17 ± 0.07; Table 2; Figure 3A). Whereas the bacterial richness associated with plant seed was not affected by the treatments, the evenness of bacterial assemblages increased *ca*. 42% by the endophyte presence (E+ = 0.50 ± 0.01, E- = 0.29 ± 0.01; Table 2; Figures 3B,C). In agreement with the endophyte-mediated effect on the evenness of bacterial communities, the relative abundances of the dominant taxonomic groups (relative abundances >1%) were more similarly distributed in endophyte-symbiotic seed compared with their non-symbiotic counterparts (Figure 4).

**Table 2:** Effect of the endophyte symbiotic status and maternal herbivory history on the bacterial composition, bacterial diversity (Shannon index, H'), bacterial richness and bacterial abundance (evenness index, J') of seed of *Lolium multiflorum* plants symbiotic or not with the fungal endophyte *Epichloë occultans*, and challenged or not with the aphid herbivore *Rhopalosiphum padi*.

		Bacterial composition (based on OTUs)			Shannon diversity index (H' of OTUs)		Bacterial richness (# of OTUs)		Bacterial evenness index (J' of OTUs)	
Treatment	*df*	*F*-values	R^2^	*p*	F-values	*p*	F-values	*p*	*F* values	*p*
Symbiosis	1,8	2.89	0.21	**0.004**	72.35	**<0.001**	0.20	0.661	106.87	**<0.001**
Herbivory	1,8	1.42	0.10	0.204	0.19	0.669	0.60	0.458	1.16	0.312
Symbiosis x Herbivory	1,8	1.56	0.11	0.157	0.58	0.466	1.36	0.276	0.08	0.782

Bacterial composition data were analysed with permutational multivariate analysis of variance (PERMANOVA). Bacterial diversity, richness, and abundance data were analysed with analyses of variance. OTUs means operational taxonomic units. Statistically significant effects (*p* < 0.050) are highlighted in bold (*n* = 3 plants).

## Discussion

We hypothesised that vertically transmitted *Epichloë* endophytes influence the composition and diversity of the seed-associated bacterial microbiota of their host plants. Furthermore, we posited that the herbivory experienced by mother plants also affect the composition and diversity of seed bacterial communities, but this effect of herbivores would interact with the presence of *Epichloë* endophytes in mother plants. Our results showed that *Epichloë* indeed influenced the composition of the bacterial communities in host seeds. The seed-associated bacterial communities were more diverse in endophyte-symbiotic than endophyte-free seeds. Instead of an increase in species richness, this higher diversity was attributed to the relative abundances among bacterial taxa being more similar in communities associated with E+ than E- seed (bacterial communities in E- seed were dominated by few taxa). Contrary to our expectation, seed bacterial communities were not affected by the herbivory experienced by mother plants.

The seed-associated bacterial communities are generally simpler in terms of composition and diversity than communities from soils, seedlings and adult plant tissues (Chesneau et al., 2020; Rochefort et al., 2021; Walsh et al., 2021). Bacteria within phylum Proteobacteria, including members from *Pantoea* spp. and *Pseudomonas* spp., normally dominate seed bacterial communities (Truyens et al., 2015; Kim and Lee, 2021). In agreement with this, bacterial communities in *L. multiflorum* seeds were dominated by members from the Proteobacteria, including the above-mentioned genera and a few other groups (e.g., *Erwinia* spp.). This pattern of bacterial diversity in *L. multiflorum* seeds is similar to those documented in seeds from other grass species such as *Lolium perenne* (Tannenbaum et al., 2020, 2021), *Elymus nutans* (Guo et al., 2021), *Oryza sativa* (Eyre et al., 2019; Wang et al., 2020a) and *Triticum aestivum* (Hone et al., 2021; Walsh et al., 2021). Several beneficial bacteria within the *Pantoea* and *Pseudomonas* genera have been isolated from plant seeds (Feng et al., 2006; Díaz Herrera et al., 2016; Verma et al., 2018). Experimental evidence has shown that beneficial seed-inhabiting *Pantoea* and *Pseudomonas* can efficiently colonise seedlings and that the colonisation of these bacteria can provide advantages to seedlings during establishment (Ferreira et al., 2008; Pavlova et al., 2017; Verma et al., 2017; Morella et al., 2019; Soluch et al., 2021).

*Epichloë* fungal endophytes can enhance the performance of host seed and seedlings (Clay, 1987; Novas et al., 2003; Stefanoni-Rubio et al., 2021). This *Epichloë*-mediated enhancement in host performance can be even more significant when symbiotic seed and seedlings experience stressful conditions (e.g., pathogens, salinity, drought, soil contamination and nutrient limitation; Gwinn and Gavin, 1992; Gundel et al., 2006; Zhang et al., 2010; Vázquez de Aldana et al., 2014; Ding et al., 2015; Pérez et al., 2016; Wang et al., 2020b). The increased performance of symbiotic seed/seedlings has been normally attributed to the biochemical changes exerted by *Epichloë* on host plants (e.g., alkaloids, antioxidants and sugars; Gundel et al., 2018; Zhang et al., 2019; Hewitt et al., 2020; Ueno et al., 2020). However, enhanced performance could also be explained, at least in part, by the beneficial activities derived from other microbial symbionts co-inhabiting *Epichloë*-symbiotic seed and seedlings. In the present study, *Epichloë* endophyte presence was associated with increased diversity and changes on composition of seed-associated bacterial communities, particularly in members from *Pantoea* and *Pseudomonas*. Interestingly, both genera contain species/strains described to provide a suite of benefits to plants such as growth promotion, nitrogen fixation, phosphate solubilisation and protection against pathogens/plant competitors (Elmore et al., 2019; Guo et al., 2021). For instance, several plant-growth promoting *Pseudomonas* isolates increased the seed germination and seedling growth of *Solanum lycopersicum* plants (through production of auxins, phosphate-solubilising compounds and another growth-promoting compounds; Qessaoui et al., 2019). The stress-protective *Pantoea alhagi* strain NX-11 bacterium increased the salt stress resistance of *Oryza sativa* seedlings (*via* exopolysaccharide production, compounds that inhibit the plant absorption of salt; Sun et al., 2020). The antimicrobial-producing *Pseudomonas* sp. strain SY1 bacterium protected seedlings of *Oryza sativa*, *Cynodon dactylon* and *Poa annua* from the fungal pathogen *Fusarium oxysporum* (Verma et al., 2018). Therefore, it is likely that the performance of symbiotic seed/seedlings can be also increased by the action of those beneficial bacteria (e.g., *Pantoea* spp. and *Pseudomonas* spp.) promoted by the association of host plants with *Epichloë* endophytes.

We did not detect an effect of the mother plant herbivory (or *via* interaction with the endophyte presence) on the seed-associated bacterial communities. In the present experiment, plants were interacting with aphids for 21 days (plants were at tillering stage), and the rest of the growing cycle were maintained free of herbivores (i.e., *ca*. 45 days). It is possible that any impact from the aphid treatment was transitory such that changes in the plant associated microbiota did not persist until the plant flowering stage when microbes are transmitted into seeds. This seems likely since herbivory triggers changes in plant defence and plant nutrient release, both of which likely impact the plant microbiota indirectly, in addition to microbes being added directly through the herbivore itself (Smets and Koskella, 2020). It is also possible that we observed minimal herbivory-associated changes on the plant’s bacterial communities because these are driven more by the colonisation of microbes from external sources such as the soil, air and biotic vectors (Tombolini et al., 1999). In fact, experimental evidence has shown that plant microbial communities can recover their pre-perturbation structures by colonisation of microbes from environmental sources (Haas et al., 2018; Stone and Jackson, 2021).

In conclusion, this study highlights the influence of *Epichloë* endophytes in the diversity of bacterial communities associated with seed. Our results indicate that these endophytes affect the seed bacterial microbiota by modifying the composition and increasing the diversity of these bacterial communities. *Epichloë* fungal endophytes may increase diversity by reducing the overall bacterial load and by preventing establishment of dominance of certain groups. It is likely that this *Epichloë*-mediated increment in bacterial diversity contributes to the documented enhanced performance of endophyte-associated seed/seedlings (e.g., Clay, 1987; Gwinn and Gavin, 1992; Pérez et al., 2016). Furthermore, due to the lack of effect of our herbivory treatment, we speculate that stresses occurring close to the plant flowering stage, when microbes are transmitted, may have larger impacts on seed-associated microbial communities than stresses acting at earlier stages (Shade et al., 2017). In fact, it has been documented that certain seed attributes in *L. multiflorum* plants can be affected by stresses occurring at, or close to, the reproductive stage (e.g., seed production and longevity; Ueno et al., 2020, 2021). Considering the relative abundances of bacterial taxa across treatments, *Kosakonia* was remarkable because this taxon exhibited high abundance and was virtually only present within E-/H- seed. Perhaps *Kosakonia* spp. were particularly susceptible to potential chemical changes exhibited by seed due to the experimental treatments. In agreement with this hypothesis, Hou et al. (2020) found that the abundance of *Kosakonia* spp. effectively depended on the plant chemical composition (in this study, *Kosakonia* spp. were also associated with only E- plants). Further experiments that isolate bacteria from *Epichloë*-associated seed will be essential to characterise the microbiota and also to identify those beneficial bacteria for plants. Sequencing of whole bacterial genomes, transcriptomes and/or inoculation of bacterial isolates into plants can be useful tools for characterising the isolated bacteria (Bastías et al., 2020; Ju et al., 2021; Li et al., 2021).

## Data Availability Statement

The data presented in the study are deposited in the NCBI Short Read Archive (SRA), accession number PRJNA782330.

## Author Contributions

DB, LB, and PG conceived the experiment. LB and AB performed the experiments. DB, RJ, IA-R, and PG analysed data. IA-R, RJ, MM-M, and PG wrote the manuscript. All authors contributed to the article and approved the submitted version.

## Funding

The research was funded by agencies FONCyT-Argentina (project PICT-2018-01593) and FONDECYT-Chile (project 1210908).

## Conflict of Interest

The authors declare that the research was conducted in the absence of any commercial or financial relationships that could be construed as a potential conflict of interest.

## Publisher’s Note

All claims expressed in this article are solely those of the authors and do not necessarily represent those of their affiliated organizations, or those of the publisher, the editors and the reviewers. Any product that may be evaluated in this article, or claim that may be made by its manufacturer, is not guaranteed or endorsed by the publisher.

## Supplementary Material

Supplementary Figure 1 Rarefaction curves of bacterial OTUs (operational taxonomic units) associated with each seed sample used in the present study.

Supplementary Figure 2 Shepard diagram of the non-metric multidimensional scaling (NMDS) ordination related to Figure 2.

Supplementary Table 1 Matrix of bacterial OTUs (operational taxonomic units) vs. read counts associated with seed samples.
