# Supplementary figures and images for: Epichloë Fungal Endophytes Influence Seed-Associated Bacterial Communities
Source: Front Microbiol. 2022 Jan 4;12:795354. doi: 10.3389/fmicb.2021.795354 (PMC8764391; doi:10.3389/fmicb.2021.795354)

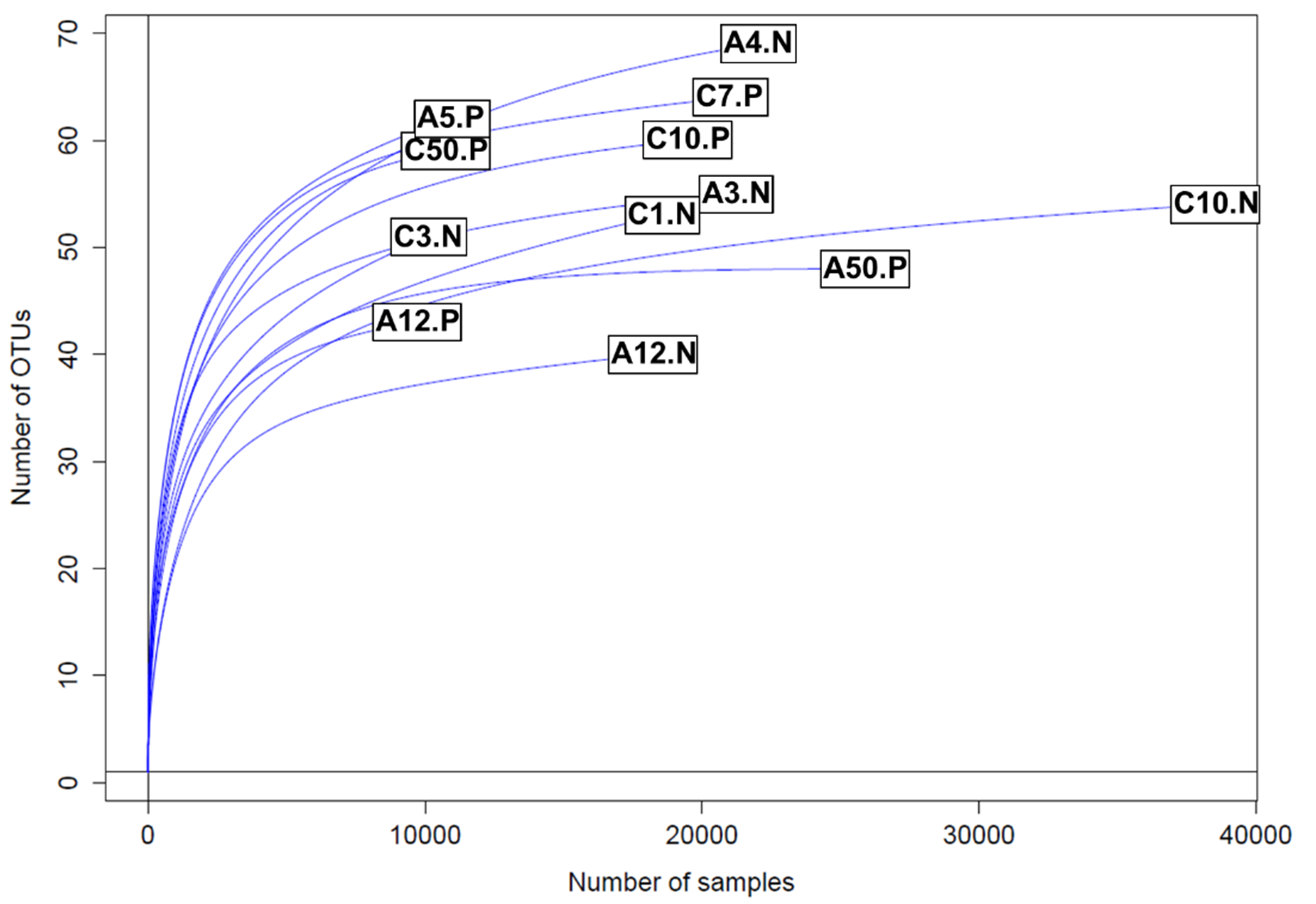

Supplement: Supplementary Figure 1 — Rarefaction curves of bacterial OTUs (operational taxonomic units) associated with each seed sample used in the present study. [file Image_1.tif]

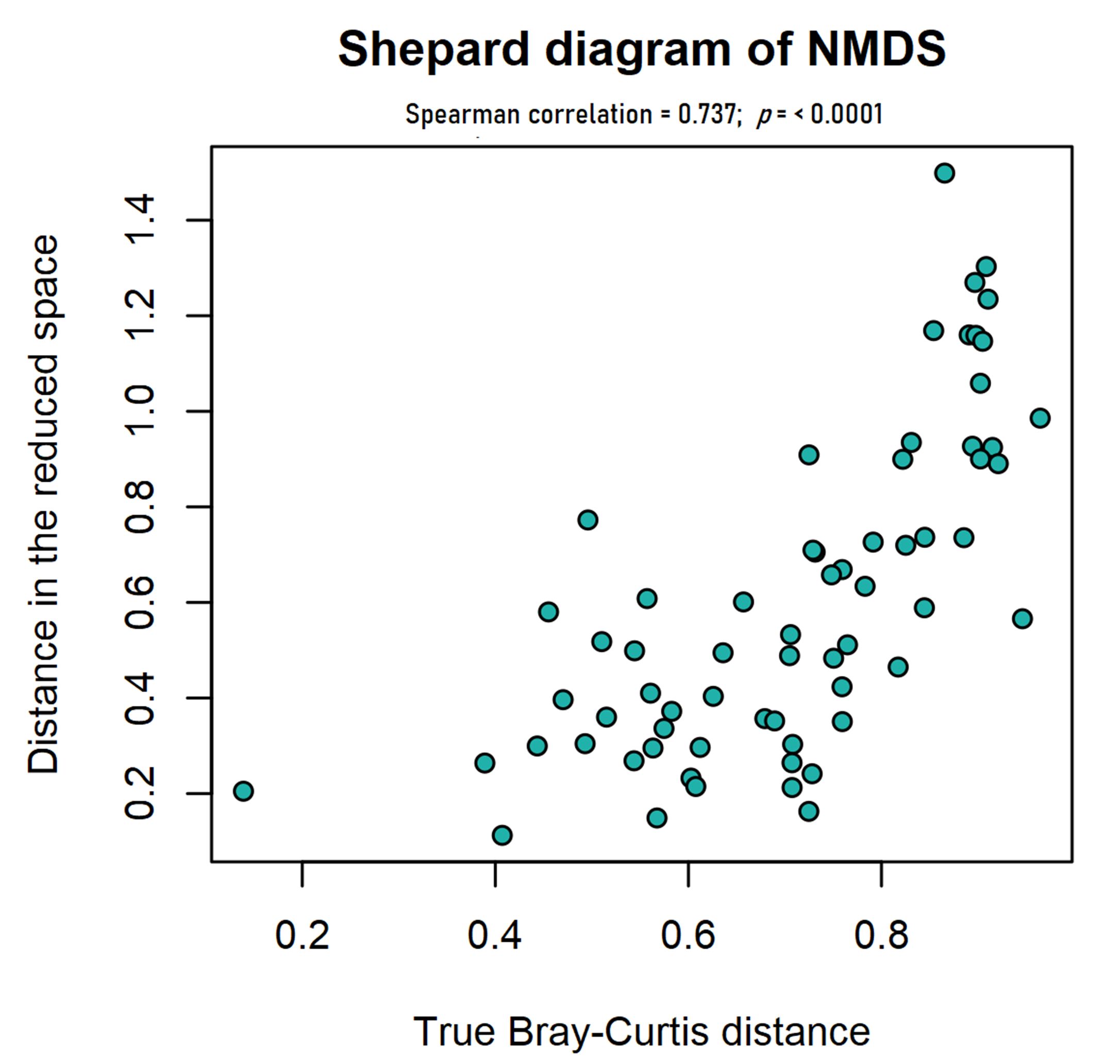

Supplement: Supplementary Figure 2 — Shepard diagram of the non-metric multidimensional scaling (NMDS) ordination related to Figure 2. [file Image_2.tif]
